# Simvastatin Loaded Dissolvable Microneedle Patches with Improved Pharmacokinetic Performance

**DOI:** 10.3390/mi13081304

**Published:** 2022-08-12

**Authors:** Zulcaif, Nadiah Zafar, Asif Mahmood, Rai Muhammad Sarfraz, Abdelhamid Elaissari

**Affiliations:** 1 Faculty of Pharmacy, The University Lahore, Lahore 54000, Pakistan; 2 Department of Pharmacy, The University of Chakwal, Chakwal 48800, Pakistan; 3 Faculty of Pharmacy, College of Pharmacy, University of Sargodha, Sargodha 40100, Pakistan; 4 Univ. Lyon, University Claude Bernard Lyon 1, CNRS, ISA-UMR-5280, F-69622 Lyon, France

**Keywords:** dissolving microneedle, transdermal drug delivery, sustained release, simvastatin, thiolated chitosan, PVP-K30, PVA, pharmacokinetic profile

## Abstract

Microneedle patches (MNPs) are one of the emerging approaches for drug delivery involving minimal invasion and improved skin penetration of macro- and micro-entities. Herein, we report dissolvable microneedle patches (dMNPs) as a novel tool for better systemic delivery of Simvastatin in the management of hypocholesteremia. Thiolated chitosan (TC), polyvinyl pyrolidone (PVP) and polyvinyl alcohol (PVA) were employed in the development of dMNPs. Developed patches were characterized through SEM, FTIR, DSC, TGA, PXRD, dissolution testing, tensile strength, elongation (%), skin irritation studies, moisture content and pharmacokinetic evaluation. dMNP F26 exhibited excellent tensile strength (9.85 MPa), penetration potential (~700 µm), moisture content (5.95%), elongation (35.54%) and Simvastatin release of 77.92%. Pharmacokinetic properties were also improved, i.e., C_max_ 1.97 µg/mL, t_max_ 9 h, MRT 19.9 h and AUC 46.24 µg·h/mL as compared to Simvastatin solution displaying C_max_ 2.55 µg/mL, t_max_ 3 h, MRT 5.91 h and AUC 14.20 µg·h/mL thus confirming higher and improved bioavailability. Kinetic modelling revealed zero order as the best fit model based on regression coefficient. Histopathological findings proved the biocompatibility of the developed dMNPs.

## 1. Introduction

Drugs can be delivered directly into the bloodstream by using various routes such as intramuscular (IM) and intravenous (IV) for the achievement of prompt therapeutic effect. However, the parenteral route of drug delivery possesses certain drawbacks. Firstly, injection involves the insertion of a hypodermic needle into the skin which causes discomfort and hence leads to lower patient compliance. Secondly, trained staff are required for the drug administration and handling. Newer, safer, effective techniques such as nano-tubing, mucosal administration, magnetically modulated drug delivery systems, nanofibers, inhalers and transdermal drug delivery systems (TDDS) have emerged to remove these reported drawbacks [1,2,3,4,5].

TDDS is a non-invasive drug delivery system in which drugs are directly delivered into blood through the skin [6]. Alejandro Zaffaroni was the first scientist who invented the transdermal patch and in 1979, FDA gave approval for the first Scopolamine-containing transdermal patch [7]. However, skin barriers affect the transport of molecules that have large molecular weight [8]. Various advancements made in this drug delivery system to solve this problem include sonophoresis, iontophoresis, electroporation, photomechanical waves, vesicles, chemical enhancer, thermal ablation, polymeric nanoparticles, nanoemulsion and microneedle patches [9].

Microneedles patches (MNPs) are micron-scale needles that administer drugs directly into the systemic circulation through the skin using a simple minimally invasive approach and offer optimum bioavailability similar to the parenteral route because of avoidance of 1st pass effect [10]. Microneedles only pass through the stratum corneum and epidermis but do not touch the nerve fibres and blood vessels in the dermis [11]. The MNPs can provide better control over the drug release and may act as a depot or reservoir system, releasing the therapeutic drug for a longer period of time [12]. Therapeutically, this kind of dosage form reduces fluctuations in plasma drug concentrations, especially for drugs that have a short half-life. MNPs are helpful to reduce the systemic side effects and increase the safety margin of the delivered drugs. These advantages lead to the administration of lower doses through microneedle patches to achieve the optimal therapeutic effect. There are different types of MNPs such as solid microneedles, coated microneedles, dissolvable microneedles and hollow microneedles [13,14,15]. Among these, dissolvable microneedles patches (dMNPs) have recently gained widespread popularity among scientists due to their low manufacturing cost, improved immunogenicity and ease of use. 

There are different ways to prepare dMNPs such as solvent casting method, wet chemical etching, solvent washing, reactive ion etching, etc [16,17,18]. In this study, the solvent casting method was selected which utilizes a number of diverse natural, biocompatible, biodegradable and synthetic polymers for the fabrication of its base. Literature shows the utilization of a vast number of polymers such as chitosan (CS), thiolated chitosan (TC), polyvinyl alcohol (PVA), carboxymethylcellulose (CMC), hydroxypropyl methylcellulose (HPMC), Alginic acid, Eudragit and polyvinylpyrrolidone (PVP), etc., for fabrication of dMNPs [19,20,21,22]. Selection of an appropriate polymer is very critical as this directly affects the mechanical properties, drug loading efficiency (%) and release behaviour from dMNPs [23]. 

CS is a nontoxic, semi-crystalline, biodegradable, and biocompatible molecule obtained after the chemical modification of chitin by deacetylation of chitin with sodium hydroxide. TC is the most important modified CS that has been reported with improved cohesion, controllable drug release and mucoadhesive properties [24]. Attachment of the thiol group to the primary amine of chitosan via amide linkages imparts superior cohesive properties and makes TC a suitable biomaterial for fabrication of dMNPs for controlled release delivery systems [25]. 

PVA is a water-soluble polymer which is biodegradable and dMNPs prepared by using PVA display superior properties as compared to other polymers (trehalose, carboxymethyl cellulose, raffinose, polyvinylpyrrolidone, sodium alginate and hydroxypropyl methyl cellulose) based dMNPs with respect to proration of the epidermis [26,27,28]. Moreover, PVA has been used in combination with other polymers such as PVP, trehalose, sucrose, carboxymethyl cellulose and dextran to fabricate drug-loaded dMNPs [29]. PVP is a biocompatible, temperature-resistant, nontoxic synthetic polymer obtained by radical polymerization of the monomer, N-vinyl pyrrolidone and shows a complex affinity for both lipophilic and hydrophilic drugs [30,31,32]. 

Statins are a group of therapeutic actives that reduce the morbidity associated with cardiovascular diseases as well as help in the reduction of cholesterol. A number of drugs fall under the class of statins such as Atorvastatin, Pravastatin, Simvastatin and Rosuvastatin [33]. Drugs which face extensive first pass effect and have lower bioavailability are the best candidates to be encapsulated in dMNPs. Among the above-mentioned drugs, Simvastatin is a drug that displays a major drawback of extensive first pass metabolism that results in very low bioavailability of around 5%. Simvastatin belongs to BCS Class-II and is extracted synthetically from the fungus Aspergillus terreus by the fermentation process. It inhibits the hepatic enzyme hydroxymethyl glutaryl coenzyme A reductase which is responsible for the conversion of hydroxymethyl glutaryl coenzyme A to mevalonate which further gets converted into cholesterol in the body so the inhibition of mevalonate results in lower cholesterol synthesis and hence, lower plasma cholesterol levels [34]. 

In this study, we report the fabrication of polymeric dMNPs for the controlled delivery of Simvastatin. The dMNPs were prepared by varying the amount of TC, PVP and PVA. Controlled release dMNPs can provide a more efficient way of administrating the drug into the bloodstream with improved bioavailability and better patient compliance. This will reduce the dosing frequency and maintain a steady plasma drug level. The formulated dMNPs were characterized and evaluated with respect to Simvastatin loading efficiency and pharmacokinetic profile.

## 2. Materials and Methods

### 2.1. Thiolation of Chitosan

Thiolation of chitosan was performed with a slight modification of the method reported by Ahmad et al. 2020 [35]. All the chemicals were precisely weighed on an electronic weighing balance. Chitosan was completely dissolved in acetic acid (2%) solution followed by the addition of thioglycolic acid (for activation of carboxylate ions) and stirring was continued until the formation of a clear solution. Then, 50 mM hydroxylamine was added to the above-formed solution and pH was adjusted to 5 by adding 1M HCl solution dropwise. 

The resultant solution was dialyzed by using a dialysis membrane five times for a period of 3 days under dark conditions in the following manner: (1) one time with 5 mM HCl solution, (2) two times with 1% NaCl containing 5 mM HCl solution to minimize ionic interactions between the cationic polymer and anionic sulfhydryl compound, (3) the final two cycles performed again by using 1 mM HCl solution. After this treatment, lyophilization was performed and the resultant product was stored at 4 °C [35]. 

### 2.2. Development of Dissolvable Microneedle Patches

Different formulations of dMNPs were developed using a solvent casting technique. A commercially available silicon molds (ST-32, array 8 × 8, height = 750 µm, base = 200 µm, pitch = 680 µm, shape = pyramid) Micropoint Technology, Singapore was utilized to prepare dMNPs. Three different polymers, i.e., thiolated chitosan (TC), polyvinyl alcohol (PVA) and polyvinylpyrrolidone (PVP) in different ratios were used to prepare different formulations (Table 1). Practically, solutions of TC, PVA and PVP were prepared separately by dissolving the specific quantity of each polymer in their respective solvents, i.e., 1% acetic acid solution, ethanol: water (50:50) mixture and deionized water, respectively. Solutions based on a single polymer as well as a combination of polymers were prepared by using vortexing for 5 min followed by stirring on a magnetic stirrer for 15 min. Centrifugation was performed on these moulds containing solution at 5000 rpm for 4 h. Next, sonication was performed for 15 min at 37 °C to fill the needles’ depth and remove the air. Sonication was followed by complete drying at room temperature for 48 h. The prepared microneedle patches were peeled off from the moulds and stored in a desiccator for further characterization.

### 2.3. Simvastatin Loading in dMNPs

Visual examination (bluntness and incomplete needle formation) helped to select 14 (F3, F4, F11–F16, F20, F21 and F23–F26) out of 26 formulations for Simvastatin loading. A stock solution (20 mg/mL) was prepared in ethanol. The drug solution was mixed with polymeric solutions of each formulation in screw-capped test tubes and mixing was continued for 24 h. Resultant solutions were sonicated for 15–20 min at 37 °C to remove any dissolved oxygen and to ensure proper mixing. After that, 1 mL of each drug-loaded solution was transferred into microneedle templates. Finally, casting was carried out to get drug-loaded dMNPs. The percentage of Simvastatin loaded into dMNP was evaluated by analyzing samples on HPLC. 

### 2.4. Optical Microscopic Evaluation of dMNPs

A light microscope (Nikon E200, Tokyo, Japan) equipped with a digital camera (DCM-35 USB 2.0 and MINISEE IMAGE) was used for the evaluation of selected 14 formulations (F3, F4, F11–F16, F20, F21 and F23–F26) appearance with respect to microneedles tips and distribution pattern of the array [36].

### 2.5. Microneedle Patch Thickness

The thickness of the selected 5 dMNPs (F4, F16, F20, F25 and F26) was measured by a digital micrometre (Mitutoyo, Kawasaki, Japan). Measurements were performed at three different points on each patch and the average thickness was calculated [37].

### 2.6. Tensile Strength and Percentage Elongation

Tensile properties of selected 5 dMNPs (F4, F16, F20, F25 and F26) were determined by using an auto tensile tester (Pram, China) in order to confirm the integrity and elongation of the patches. Developed patches were placed between two fixed jaws of the tester and they were mechanically allowed to move apart from each other. The initial and final length of the patches was noted and the percent elongation was calculated by using given below equation:

(1) $$Elongation\;\left(\%\right)=LP_{f/}LP_{I}\;\times \;100$$

where, *LP_f_* and *LP*_I_ are the final and initial lengths of the developed patch at breakage [37]. 

### 2.7. Moisture Contents (%)

The moisture content of the selected 5 dMNPs (F4, F16, F20, F25 and F26) containing Simvastatin was determined by a moisture analyzer (Sartorius, Gottingen, Germany) to check the integrity of dMNPs [35].

### 2.8. Scanning Electron Microscopy

Surface morphology, needle shape, needle size, needle base and needle pitch of selected dMNP (F26) were determined by using SEM (NOVA Nano SEM 450, FEI, Columbia, SC, USA) at different magnification values [38]. 

### 2.9. In Vitro Penetration Study

The dMNP (F26) penetration efficiency was studied using Parafilm M^®^. The film was folded into eight layers without any stretching. Folded parafilm was placed on a glass slide. The dMNP was placed onto the parafilm M with gentle pressing. The paraffin film was then unfolded, and the depth of penetration was examined under a light microscope [39].

### 2.10. Fourier Transform Infrared Spectroscopy

FTIR studies are usually carried out to confirm the compatibility of the ingredients, complex formation and confirmation of functional groups prior to and after the fabrication of patches. FTIR (ATR-FTIR, Bruker, Billerica, MA, USA) was operated to record spectra of TC, PVA, PVP, Simvastatin, and drug-loaded dMNP. FTIR spectroscopy was operated over a specific scanning range of 400–4000 cm^−1^ [35,37,40].

### 2.11. Differential Scanning Calorimetry

This technique was used to record enthalpy changes and phase transition behaviour of TC, PVA, PVP, Simvastatin and the prepared dMNP against increasing temperature. Every ingredient was heated up to 300 °C at a heating rate of 10 °C min^−1^. The instrument was validated before analysis [35,37,41].

### 2.12. Thermogravimetric Analysis

Thermogravimetric analysis was performed to confirm weight loss against increasing temperature. The samples including TC, PVA, PVP, Simvastatin and drug-loaded dMNP were heated from 25 °C to 350 °C at a heating rate of 10 °C min^−1^. The loss in weight was measured to observe any changes in the thermal ability of the dMNP [42].

### 2.13. Powder X-ray Diffraction Studies

X-ray diffraction is performed to confirm the nature of the ingredients, i.e., crystalline or amorphous. The analysis was conducted in 2*θ* mode on samples using a bracket holder for samples with a source of radiation, i.e., Cu Kα (1.54059 Å) at room temperature. Data were recorded between 2*θ* values of 10° and 60° by using a step size of 0.013° and an acquisition time of 30 s per step. Analysis (TC, PVA, PVP, Simvastatin and drug-loaded dMNP) was at 40 mA and 45 kV. XRD diffractograms were analyzed [43].

### 2.14. Skin Irritation Studies of dMNPs

A selected microneedle patch of Simvastatin (F26) was applied on rabbit skin to check the occurrence of any erythema/edema by using Draize scoring. Hairs of the dorsal surface of the rabbit were trimmed using an electrical trimming machine (Kemei, Nanchang, China), wiped with phosphate buffer (pH 7.4) and shaved. One side of the dorsal surface of rabbit skin was kept as control and on the other side dMNP (F-26) was applied for 48 h. Skin condition was observed and compared regarding erythema/edema and irritation/redness [37,44].

### 2.15. Simvastatin Loading Efficiency (%)

The amount of drug loaded in the microneedle patch was determined carefully by separating the needles from the base plate using a sterile surgical blade (Feather Safety Razor Co., Ltd., Seki, Japan) under an optical microscope. The detached needles were dissolved in Acetonitrile:Phosphate buffer (65:45) by vortexing and ultra-sonication for 30 min. For the determination of drug concentration, samples were analyzed by HPLC through the method reported earlier by Dey et al., 2013 [45]. Blank microneedles were also processed in a similar manner and tagged as control.

### 2.16. Histopathological Examination

This study was performed to evaluate any histopathological event occurring upon application of dMNPs on rabbit skin. The skin was obtained from the dorsum part of the rabbit. The skin fats were removed by using a surgical blade (Feather, Seki, Japan) and the skin was placed on the previously moistened (phosphate buffer pH 7.4) filter paper to provide mechanical support. One side of the dorsum was treated as control and on the other stratum corneum side dMNP was applied with a gentle pressure of thumb for 1 min. dMNP was removed and processed by staining technique to evaluate and compare general morphology with control [46]. Both parts of the skin (control and treated) were separately stained by hematoxylin and eosin staining through the following procedure [47]. The skin was immersed in 10% formalin solution and then in acetone + 10% formalin solution for 4 h in a tissue processor (rotary). Liquid wax was obtained from a tissue embedding station (Shandon, Histocenter, Thermo electron cooperation, Waltham, MA, USA) in a block-making dye and skin was placed in it. It was dried for 3 h at room temperature and then skin-loaded wax was placed in a microtome (Thermo Scientific, Waltham, USA) for slicing to prepare the slide for microscopic examination. Then this slice was dipped in water where the temperature was kept at 37 °C. Glass slide was used for lifting the prepared slice of tissue on the slide. The slide was covered with a coverslip. This prepared slide was placed in a stain rack and staining was performed by dipping the slide in different dyes and chemicals in the following sequence: in Xylene solution twice for 2 min, in ethanol thrice for 2 min, in water for 2 min, in hematoxylin for 2 min, in water for 10 min, in eosin for 1 min and at the end dipped for 1 min in acetone. Finally, the slide was observed microscopically for any histological changes in the tissue layers [48].

### 2.17. In Vitro Release Study

In vitro release testing profile was performed by Franz diffusion cell with a receptor compartment having a volume of 7.5 mL and diffusion area of 2.5 cm^2^. Rabbit abdominal skin was placed in between the receptor and donor compartment of the Franz diffusion cell in such a way that the stratum corneum side faces the donor compartment. The prepared Simvastatin suspension and dMNPs were placed over the skin separately. Phosphate buffer (pH 7.4) was placed in the receptor compartment of the Franz cell. The temperature was kept at 37 ± 1 °C and the whole assembly was placed on a magnetic stirrer. Then, 0.5 mL of samples was withdrawn at fixed time intervals. An equal volume of phosphate buffer (pH 7.4) was introduced into receiving compartment after each sampling. Simvastatin content present in each sample was evaluated through the HPLC method [45].

### 2.18. Pharmacokinetic Evaluation 

The reported HPLC method for Simvastatin quantification in plasma samples with slight modifications was used. HPLC (Shimadzu, Kyoto, Japan) system equipped with UV-detector and C-18 column (5 µm × 4.6 mm × 250 mm) was employed for the acquisition of chromatographs [45]. Acetonitrile: Phosphate buffer (65:45) was used as a mobile phase whose pH was maintained by adding a few drops of Orthophosphoric acid. The system was operated at 1 mL/min flow rate. Analysis of samples was performed at 238 nm. A total of eighteen albino rabbits (2–2.3 kg) were used in this study. Rabbits were kept under light and dark cycles for a period of 7 days for acclimatization purposes. A crossover study design was used. Rabbits were separated into three groups (*n* = 6), i.e., Group A (Control), Group B (treated with marketed tablet Simva 20 mg) and Group C (treated with dMNPs containing 20 mg Simvastatin). Rabbits were kept on fasting for 12 hrs but were not deprived of water. For quantification purposes, 3–4 mL samples of blood were withdrawn from rabbits (jugular vein) at fixed time intervals up to 24 h. Every sample was transferred to EDTA tubes and centrifugation was performed at 5000 rpm for 10 min so that plasma could be separated. Then, 1 mL of plasma was subjected to deproteinization by the inclusion of an equal volume of HPLC scale methanol as a protein precipitant. Complete mixing of plasma and protein precipitant was achieved by vortexing the mixture for 3 min. Centrifugation was carried out for 15 min for the complete possible removal of plasma proteins. The supernatant was carefully collected by using a micropipette and injected into the HPLC system. After a washout period of two weeks, Group B was administered dMNPs containing 20 mg Simvastatin and Group C was administered marketed tablet Simva 20 mg. The entire procedure was repeated as per the requirements of the crossover study design. Different aspects of pharmacokinetic profile, i.e., C_max_, t_max_, AUC_0–t_, V_d_, AUM, AUC_0–∞_, and t_1/2_ were determined by analyzing data through Excel-based Adds in the program, i.e., pK solver.

### 2.19. Statistics 

Each characterization was performed trice and mean ± S.D was calculated. The statistics were applied using Graph Pad Prism software, keeping the level of significance at *p <* 0.5.

## 3. Results and Discussion

### 3.1. Thiolation of Chitosan and Development of Dissolvable Microneedle Patches

Chitosan was successfully thiolated into TC. A definite number of thiol groups and disulfide linkages were noted in TC. Results were reported in our previous study [35]. A total of 26 formulations (Table 1) were developed by using the solvent casting technique in order to obtain the best dMNP formulation with the desired outcomes. PVA and PVP are hydrophilic polymers and offer poor mechanical properties to the systems where added and TC offer better mechanical strength to dMNP when co-processed with these polymers. Variable contents of TC, PVP and PVA were tried in these 26 prepared formulations in order to design and finalized a stable formulation with desired release potential, painless pricking, microneedle shape, mechanical properties, etc. 

### 3.2. Optical Microscopic Evaluation of dMNPs

**Figure 1:**
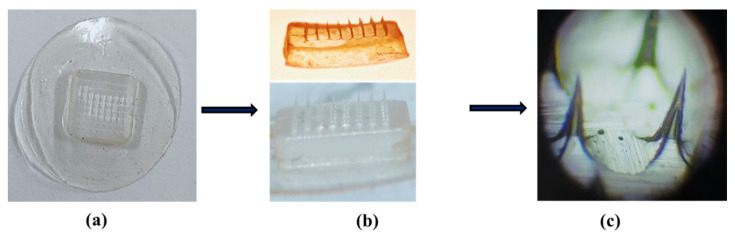
Microneedle patch F26, (**a**) macroscopic aerial view, (**b**) macroscopic side view and (**c**) microscopic view showing clear pointed needles.

When observed under a light microscope, only 5 formulations (F4, F16, F20, F25 and F26) out of 14 (F3, F4, F11–F16, F20, F21, F23–F26) displayed uniform surface, similar geometry and good sharpness of needles (Figure 1). Moreover, these screened dMNP formulations were transparent with each having 64 microneedles (8 × 8 arrays) in each formulation. In the rest of the formulations, needle sharpness and geometry were not up to the mark. 

### 3.3. Microneedle Patch Thickness

**Figure 2:**
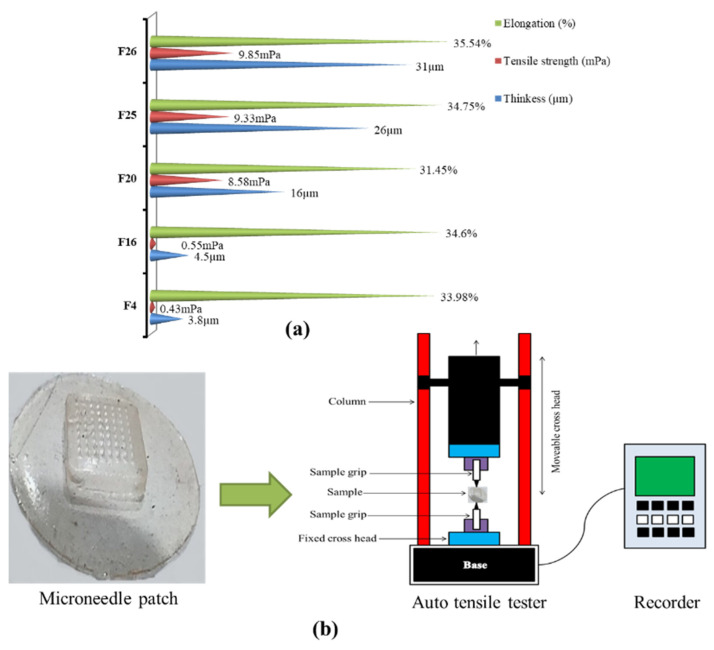
(**a**) Results of thickness, tensile strength and elongation (%), (**b**) schematic diagram to represent measurement of tensile strength and elongation of dMNP.

Patch thickness was found to be dependent upon the concentration of polymers. The thickness affects the mechanical properties of the patch as well as the distribution of drug contents within the patch. Thickness was measured from different points within an individual patch and was found to be uniform in all areas. The average thickness of the selected samples, i.e., F4, F16, F20, F25 and F26 was measured (selection based on optical microscopy results) and it was found to be 3.8, 4.5, 16, 26 and 31 µm, respectively as shown in Figure 2a. Based on the thickness results, formulation F26 was chosen as the best one as it was displaying uniform drug loading and distribution without any loss in contents during fabrication as well as having appropriate mechanical strength.

### 3.4. Tensile Strength and Percentage Elongation

Elongation (%) and strength were ascertained in order to verify the mechanical profile of the developed dMNPs. Percentage elongation confirmed the elastic nature of the dMNPs. It was noted that formulations having higher elongation possessed significant elasticity. Moreover, these two parameters are associated with the handling and application of patches onto the skin. The average tensile strength and elongation (%) of formulations F4, F16, F20, F25 and F26 were found to be 0.43 mPa, 0.55 mPa, 5.58 mPa, 9.33 mPa, 9.85 mPa and 33.98%, 34.6%, 31.45%, 34.75% and 35.54%, respectively as shown in Figure 2a. Out of these five formulations, F26 exhibited optimum tensile strength and elongation (%) because of the optimum concentration of PVA along with TC. This improvement of tensile strength could be due to the chemical interaction of hydroxyl groups of PVA and amino groups of TC that result in a compact and firm structure of the polymeric network. Likewise, disulfide linkages and thiol groups of TC also promote mechanical properties and uptake of the solvent, respectively. A rise in tensile integrity with an increase in PVA contents has already been reported in a study conducted by Dathathri et al. (2019) [49]. Moreover, the presence of PVP-K30 imparted significant elasticity and smoothness to the needles of dMNP. A schematic diagram to represent the measurement of tensile strength and percentage elongation is shown in Figure 2b.

### 3.5. Moisture Contents (%)

The moisture content (%) of selected formulation F26 was found to be 5.95% ± 0.79. Higher moisture contents result in poor integrity and mechanical properties of developed dMNPs. If moisture content is too low it can lead to brittleness in dMNPs. 

### 3.6. Scanning Electron Microscopy

**Figure 3:**
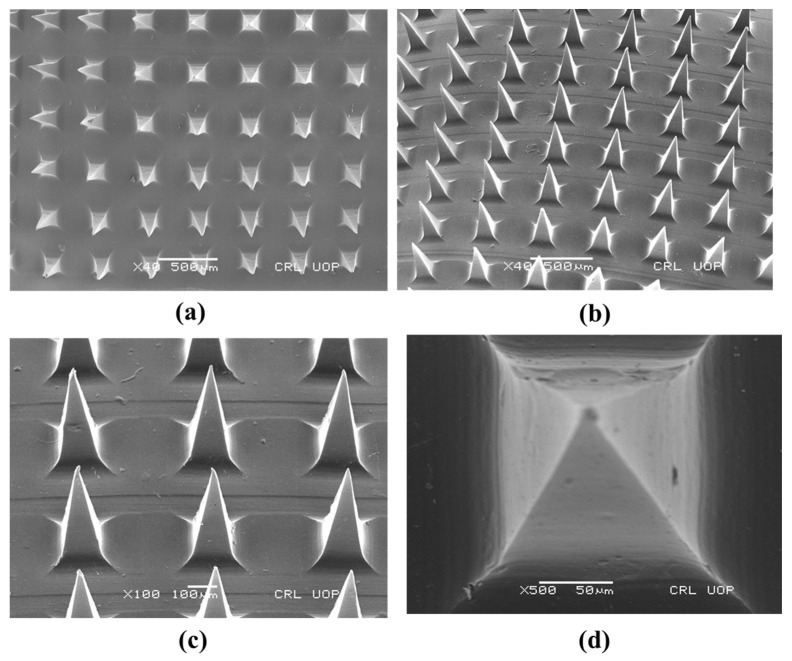
SEM photomicrographs of F26 at different magnification powers (**a**) ×40, (**b**) ×40 (aerial view), (**c**) ×100 (side view) and (**d**) ×500.

SEM analysis was performed to observe the topography, consistent distribution of microneedles and dimension of needles of the prepared dMNPs. Figure 3a–c presented that optimized polymeric dMNP (F26) possessed sharp-tipped needles, pyramidal in shape and smooth surface. The needles were homogeneous and displayed a smooth surface. Figure 3d showed that the individual needle was intact with a height of 700 µm and a base width of 200 µm indicating the successful development of dMNP. Needle to needle distance or pitch was 680 µm. Obtained dimensions also showed that the prepared microneedles could efficiently cross the skin for the delivery of the drug. Similar results were obtained by Harvinder and Mark 2007. They prepared coated microneedles with a length of 700 µm which displayed good delivery of the simvastatin into the skin [50].

### 3.7. In Vitro Penetration Study

**Figure 4:**
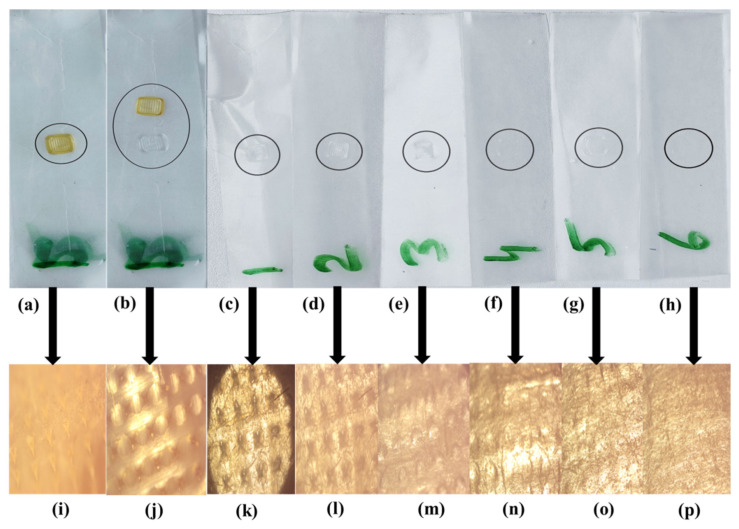
Post insertion microscopic view of the layers, (**a**) microneedle patch applied on paraffin M film 09 layers, (**b**) MNP removed from layers, (**c**) 1st layer of paraffin M film, (**d**) 2nd layer of paraffin M film, (**e**) 3rd layer of paraffin M film, (**f**) 4th layer of paraffin M film, (**g**) 5th layer of paraffin M film, (**h**) 6th layer of paraffin M film, (**i**) sharp microneedles applied on layers, (**j**) after MNP insertion microscopic view, (**k**) microscopic view of 1st layer, (**l**) microscopic view of 2nd layer, (**m**) microscopic view of 3rd layer, (**n**) microscopic view of 4th layer, (**o**) microscopic view of 5th layer, (**p**) microscopic view of 6th layer with absence of microneedle mark.

Selected dMNP (F26) was applied to pre-folded parafilm M by applying pressure on the patch backing with the help of the thumb. The average thickness of one layer of parafilm M was 0.140 mm (~140 µm) and developed dMNPs pierced five layers (~700 µm) of parafilm M. More than 80% of microneedle tips retained their geometry while few the microneedle fine tips parts were found in parafilm M. Microscopic examinations of parafilm exhibited projections of the microneedles into film thus confirming the fact that the developed microneedles will successfully penetrate across the stratum corneum (~150–200 µm) for drug delivery. This may lead to a pronounced pharmacological response due to a rise in bioavailability of Simvastatin. Results are shown in Figure 4 and Table 2.

### 3.8. Fourier Transform Infrared Spectroscopy

**Figure 5:**
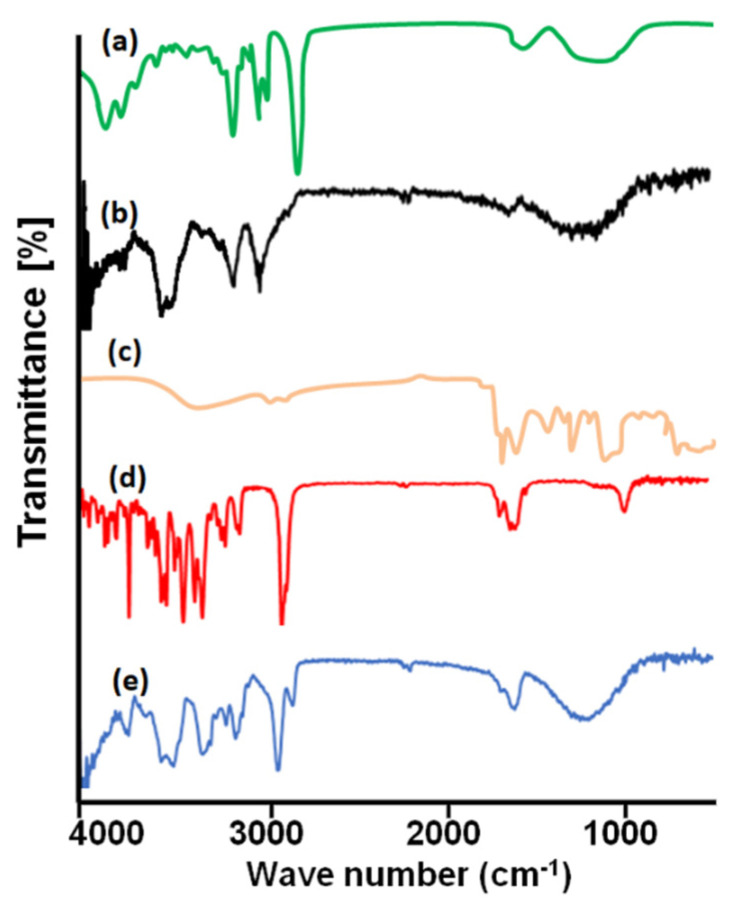
FTIR Spectra of (a) PVP-K30, (b) PVA (c) Thiolated Chitosan, (d) Simvastatin and (e) Simvastatin loaded dMNP.

FTIR was performed to verify the compatibility of the formulation ingredients, new complex formation and to confirm the existence of functional groups. IR spectrum of PVP (Figure 5a) was recorded. It exhibited a wide band at 1642.13 cm^−1^ due to stretching vibrations of the C=O bond of the pyrrolidone functional group. The peak at 2981.67 cm^−1^ belonged to the asymmetric stretching of the CH_2_ group. The peaks at 1290.14 cm^−1^ and 1425.13 cm^−1^ were due to CH group deformation. Peaks at 1313.11 cm^−1^ corresponded to C-N bending movements. FTIR spectrum of PVA exhibited evident bands at 3296 cm^−1^, 2921 cm^−1^, 1411 cm^−1^ and 1090 cm^−1^ due to –OH stretching, –CH stretching, –OH deformation and –C-O- stretching, respectively (Figure 5b). FTIR spectrum of thiolated chitosan (TC) presented typical peaks at 3352.5 cm^−1^ and 3201 cm^−1^ corresponding to –OH and –NH stretching, respectively. A new peak due to the acylamino group emerged at 1631 cm^−1^ confirming the successful conversion of chitosan to TC. The intensity of the peak at 1605 cm^−1^ was reduced due to the conjugation of the amino group with thioglycolic acid (Figure 5c). FTIR spectrum of Simvastatin showed peaks at 3550 cm^−1^ and 3747 cm^−1^, 2950 cm^−1^, 1740 cm^−1^, 1161 cm^−1^ and 1063 cm^−1^ due to –OH stretching, –CH stretching vibration, –C-O- stretching vibration of the carbonyl group and –C=O stretching vibration, respectively (Figure 5d). FTIR analysis of Simvastatin loaded dMNP (F-26) exhibited different peak patterns as compared to IR spectra of pure ingredients. Simvastatin peak at 1740 cm^−1^ due to –C-O- stretching was shifted to 1644.35 cm^−1^. The intensity of the peak at 2950 cm^−1^ due to –CH stretching was reduced (Figure 5e). Results are also computed in Table 3. Peak shifting and variation in intensities of peaks confirmed the successful fabrication of dMNP. 

### 3.9. Differential Scanning Calorimetry

**Figure 6:**
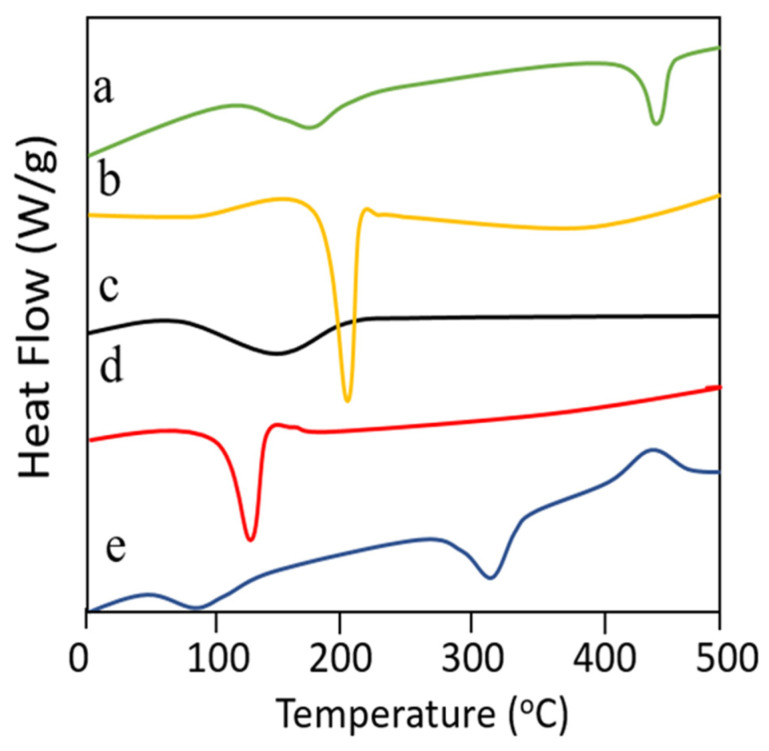
DSC thermograms of (a) PVA, (b) thiolated chitosan, (c) PVP-k30, (d) Simvastatin and (e) Simvastatin loaded dMNPs.

Enthalpy changes in the polymer(s), Simvastatin and the prepared microneedle patch were recorded by differential scanning calorimetry (DSC). DSC thermogram of PVA (Figure 6a) depicted initially an endothermic peak due to loss of moisture followed by an endothermic event starting from ~192 °C and ending at near 210 °C thereby representing phase transition, i.e., from solid to liquid. Final degradation occurred above 400 °C indicating complete combustion of the polymer structure. DSC thermogram of thiolated chitosan (TC) (Figure 6b) represented a sharp endothermic peak near 231.22 °C due to the existence of side polymeric chains attained after thiolation of chitosan. DSC analysis of PVP-K30 revealed the absence of any typical endothermic or exothermic peaks in its DSC thermogram (Figure 6c) except a flat slight wide curve indicating its transition thereby confirming its non-crystalline nature also. DSC results of pure Simvastatin depicted an evident endothermic peak at 137.12 °C due to the melting of this drug (Figure 6d). DSC findings of developed Simvastatin loaded dMNP confirmed the presence of an initial endothermic peak near 100 °C due to moisture loss followed by another endothermic above 328 °C reflecting phase transition. Finally, an exothermic peak was seen at above 440 °C due to the complete combustion of the patch (Figure 6e).

### 3.10. Thermogravimetric Analysis

**Figure 7:**
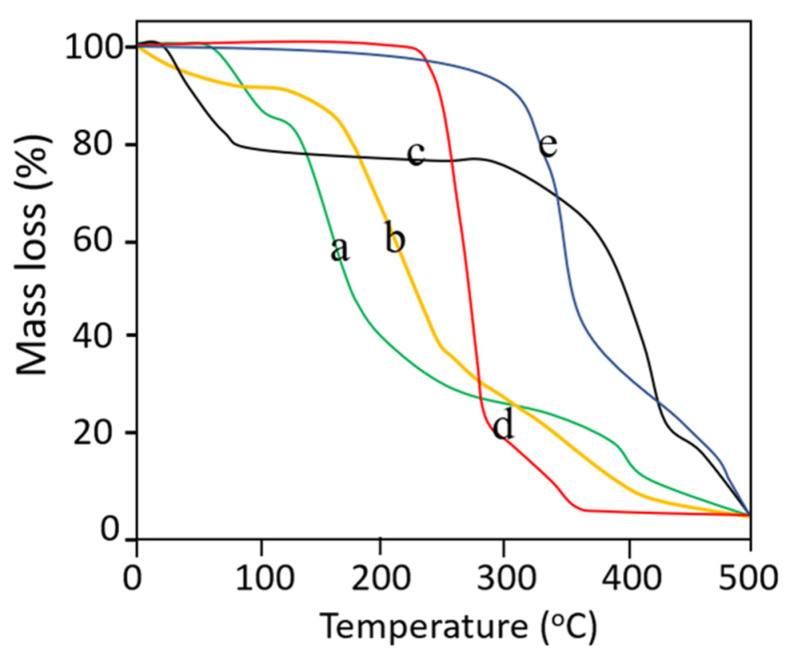
TGA thermograms of (a) PVA, (b) Thiolated Chitosan, (c) PVP, (d) Simvastatin and (e) Dissolvable microneedle patch.

TGA studies on pure ingredients and selected dMNP were conducted in order to confirm % mass loss against increasing temperature. TGA thermogram of PVA exhibited initial mass loss (~4.16%) event below 100 °C due to loss of moisture content. Moisture loss was followed by further major mass loss (~79.16%) at 250 – 400 °C which is related to the removal of hydroxyl groups in the form of water, aldehydes, and methyl ketones. Final mass loss (~97.31%) occurred at above 400 °C which was due to complete combustion of the PVA thus leaving behind carbon and hydrocarbons. Results are presented in Figure 7a. The thermogram of PVP showed two-step degradation. Initially, the first mass loss (~18.76%) was observed at 100 °C. A major degradation event occurred at 320–460 °C involving weight loss of 76.83% which represented the complete decomposition of PVP. Results are shown in Figure 7b. TGA thermogram of thiolated chitosan showed three steps of degradation, i.e., in the first step, mass loss of 10–12% occurred at a temperature range of 100 to 210 °C. Maximum mass loss (~41.32%) occurred at 220–270.20 °C. The final mass loss of 30.23% occurred at 290.12–410.43 °C. Results are shown in Figure 7c. In the case of the TGA thermogram of Simvastatin, maximum weight loss (~77.15%) occurred from 250.12–291.12 °C and the second phase of degradation with further mass loss of nearly 19.76% occurred above 310 °C. Results are shown in Figure 7d. In the TGA thermogram of dMNPs entirely different results were observed as compared to individual contents. Initial 10.13% mass loss occurred at 100–360.44 °C followed by a mass loss of 40.21% at 370.25 to 395.21 °C. At 480.12 °C intact mass was 10% which highlighted the stability of developed dMNP at elevated temperatures. Results are depicted in Figure 7e.

### 3.11. Powder X-ray Diffraction Studies

**Figure 8:**
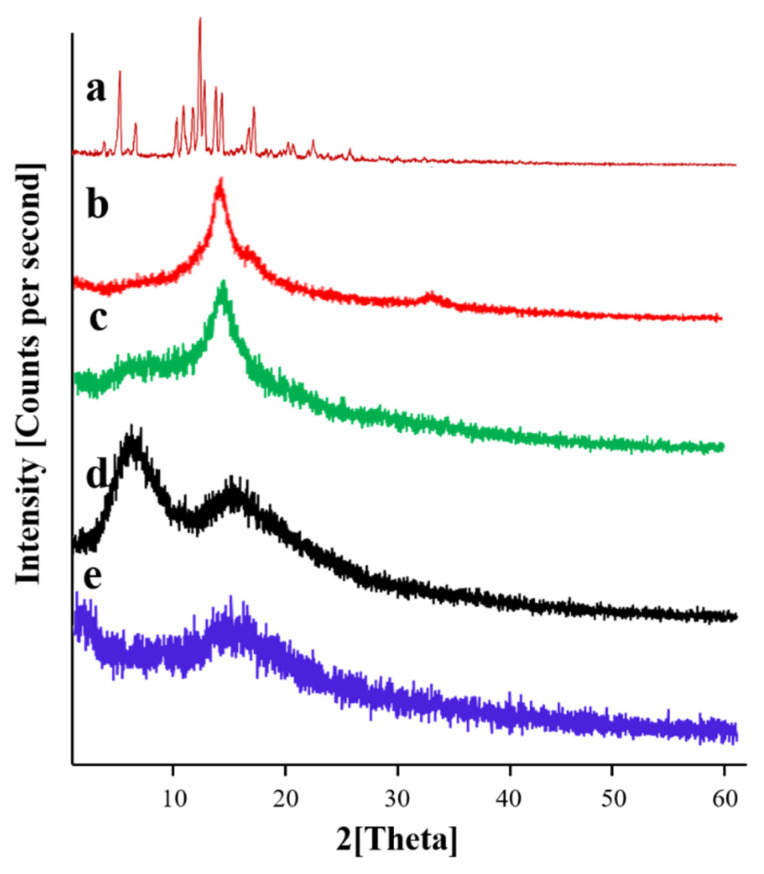
PXRD diffractograms of (a) Simvastatin, (b) PVA, (c) Thiolated chitosan, (d) PVP and (e) Simvastatin-loaded dMNPs.

PXRD analysis was performed to validate the crystalline and amorphous nature of the pure ingredients and Simvastatin loaded dMNP (F26). PXRD diffractogram (Figure 8a) of Simvastatin exhibited intense and evident peaks at 9.28°, 10.84°, 14.82°, 15.44°, 16.42°, 17.14°, 18.8°, 19.30°, 22.5°, and 28.3° thus confirming its crystalline nature. PVA diffractogram (Figure 8b) exhibited two evident and sharp peaks at 19.72° and 40.22° thus proving its crystalline nature. Likewise, the diffractogram of TC (Figure 8c) exhibited a sharp and prominent peak at 19.98°. PXRD diffractogram of PVP (Figure 8d) showed two broad peaks at 10.82° and 20.48°. The peaks were not sharp and were fused thus depicting the semi-crystalline nature of PVP. No sharp and clear peaks were seen in the diffractogram of Simvastatin-loaded dMNP (Figure 8e). All the distinct peaks displayed by the Simvastatin diffractogram were transformed into fused peaks in loaded dMNP. This observation employed that Simvastatin was converted to an amorphous form with a better solubility profile in the patch.

### 3.12. Skin Irritation Studies of dMNPs

**Figure 9:**
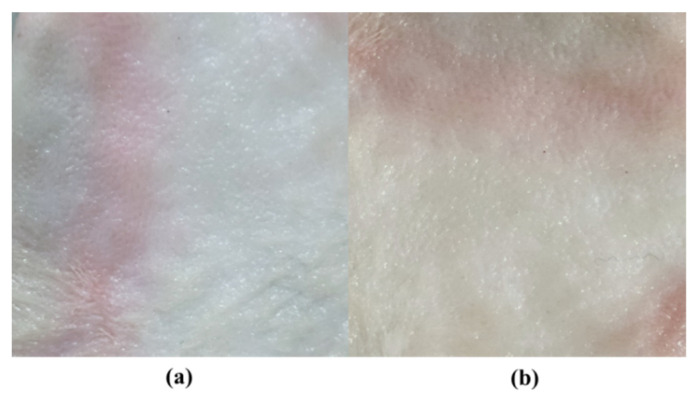
Irritation testing after application of dMNP, (**a**) Microneedle patch loaded with Simvastatin on skin before application at 0 h, (**b**) After 48 h of application.

A microneedle patch was applied to rabbit skin in order to check any sort of irritation by using the Draize scoring method. Before the application of dMNP, rabbit skin was made hairless by applying depilatory cream (Anne French^®^, Unilever, Karachi, Pakistan). Observations were recorded at 0 and 48th h. Slight erythema was noted upon applying dMNP (Figure 9a). This may be due to the higher number of microneedles patches resulting in pores on the skin. However, after 48 h, no signs of skin allergy, erythema or edema were recorded (Figure 9b). These findings suggested that the developed dMNP was biocompatible, nontoxic and non-irritant at the site of application. 

### 3.13. Simvastatin Loading Efficiency (%)

The amount of Simvastatin loaded into needles of dMNP was evaluated through the HPLC method. The loading efficiency of Simvastatin within the needle was found to be 92.37% (F26). These findings depicted the suitability of the developed formulations and methods being used in the fabrication of dMNPs. It also reflected simvastatin stability and excellent content uniformity within the developed dMNP. Moreover, loading efficiency could be tuned by varying the Simvastatin quantity in the drug stock solution. 

### 3.14. Histopathological Examination 

**Figure 10:**
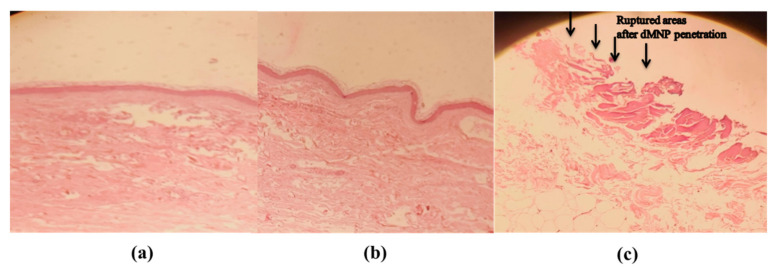
Histopathological examination of rabbit skin (**a**) before application of dMNP (**b**) after application of dMNP for 30 s and (**c**) after application of dMNP for 1 min.

Histopathological examination along with penetration ability of dMNPs through rabbit skin was investigated. The dMNP was placed onto the skin with slight pressure. The effect of variable pressure time (30 s, 01 min) was also observed. Histopathological examinations before applying dMNP revealed the absence of any invagination and pores in the superficial dermis (Figure 10a). In the case where pressure was applied for 30 s, invaginations were observed which reflected that dMNP had made contact with the superficial dermis (Figure 10b). Whereas on the other hand, when pressure was increased, rupturing of skin occurred as indicated by small arrows (Figure 10c). This fact was confirmed by observation of evident pores under a microscope after H & E staining. These results confirmed that the needles could pierce the subcutaneous layer of rabbit skin and reach the dermis. Microscopic examination of histopathological slides confirmed insertion of needles into the skin up to 625 µm of depth into the viable epidermis. Li et al. 2019 observed similar kinds of results [48].

### 3.15. In Vitro Release Study

**Figure 11:**
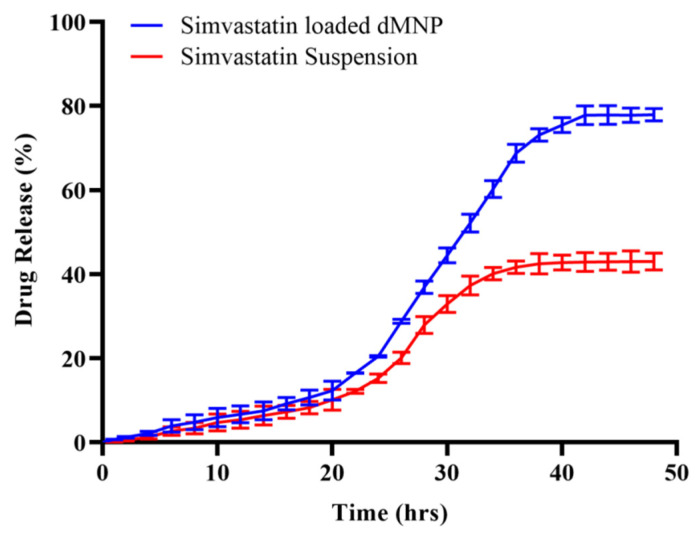
In vitro release of Simvastatin loaded dMNP and Simvastatin solution at pH 7.4.

In vitro release was assessed through rabbit skin for the best formulation F26. This study was executed in phosphate buffer (pH 7.4) by using a Franz diffusion cell. The percentage contents of Simvastatin permeating through the rabbit skin was found to be 77.92% ± 1.25 as compared to Simvastatin suspension (43.12% ± 1.75), after administration of equal quantities of Simvastatin in both cases (Figure 11). Moreover, our results were comparable to previous studies with respect to drug release behavior (Table 4).

Release data were fitted into kinetic models, i.e., zero order, first order, Higuchi and Koresmeyer Peppas model to verify the model that best fit the release kinetics. Based on the results of regression coefficient R^2^ (0.989), the zero-order kinetic model was declared the best fit model. While processing release data through the Koresmeyer Peppas model, the value of R^2^ was 0.977 with the value of n = 1.436 (Table 5) confirming Super case II release as the mechanism of release of Simvastatin from dMNPs. Super case II release is associated with chain relaxation due to contact with water or dissolution media. However, in our case, Super case II transport may be due to dissolution of PVP resulting in channelling and thereby promoting dissolution of PVA [35,54].

### 3.16. Pharmacokinetic Evaluation

**Figure 12:**
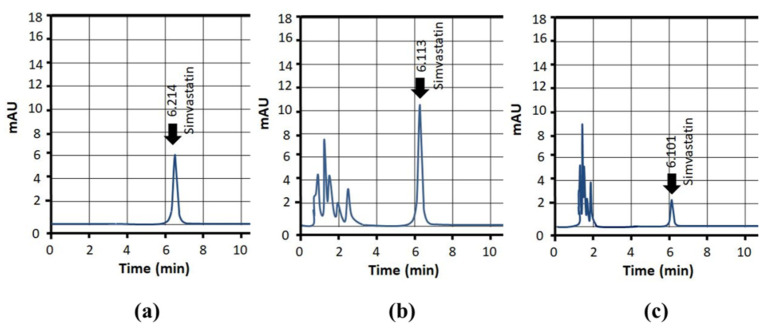
HPLC chromatographs of Simvastatin, (**a**) in mobile phase, (**b**) in spiked plasma and (**c**) after application of dMNP.

To estimate Simvastatin concentration within blood plasma, a standard curve was constructed from different known concentrations of Simvastatin. Eight different dilutions, i.e., 1, 3, 6, 12, 24 and 48 µg/mL were prepared, and a standard curve was plotted from the absorbance values of these data. Absorbance response was linear over the studied range. The value of the coefficient of regression was 0.998. Typical HPLC chromatograms of Simvastatin in the mobile phase, plasma and after administration of dMNP were recorded and presented in Figure 12.

**Figure 13:**
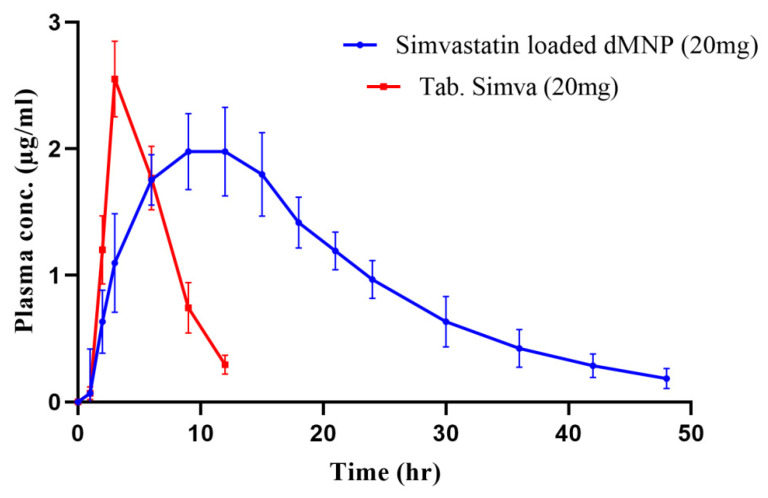
Plasma concentration vs. time graph.

In vivo pharmacokinetic study was carried out on healthy rabbits to evaluate sustained release potentials of Simvastatin loaded dMNP and to compare the pharmacokinetic profile (t_max_, C_max_, t_1/2_, AUC, MRT and bioavailability) of dMNP F26 with the marketed tablet Simva. Simvastatin concentrations measured from microneedles and Table Simva at different time intervals from the plasma concentration versus time curve are depicted in Figure 13. Maximum concentrations (C_max_) of Simvastatin released from oral tablets and dMNPs were found to be 2.55 µg/mL and 1.97 µg/mL, respectively. The time to achieve maximum plasma concentration (t_max_) was 3 h in the case of tablets thus confirming rapid absorption. While in the case of dMNP, the time to achieve maximum plasma concentration (t_max_) was increased up to 9 h. The half-life of Simvastatin in the case of the tablet Simva was 2.31 h which was increased up to 10.1 h in the case of Simvastatin-loaded dMNP. An increase in half-life indicated the slow release of the Simvastatin leading to prolonging the stay of the drug within plasma thus contributing to markedly enhanced bioavailability and sustained effect of Simvastatin in plasma. In addition to this, the mean residence time (MRT) and area under the curve (AUC) of tablet Simva (5.91 h and 14.20 μg·h/mL) were also increased in dMNP (19.91 h and 46.24 μg·h/mL), respectively. A significant difference was noted within the pharmacokinetic profile of the Simva tablet and developed Simvastatin-loaded dMNP (Table 6). Hence, in vivo evaluation of optimized dMNP revealed the suitability of dMNP for prolonging the systemic availability of Simvastatin.

## 4. Conclusions

Simvastatin-loaded dMNPs were successfully prepared as well as characterized. dMNP (F26) consisted of dimensions 8 × 8 and 64 microneedles with insertion capacity up to ~700 µm in Parafilm M. The patch displayed a Simvastatin release profile for 48 h indicating a sustained release effect. This would greatly enhance patient compliance. The improved pharmacokinetic profile, i.e., C_max_ (1.97 µg/mL), t_max_ (3 h), MRT (19.91 h), AUC (46.24 μg·h/mL) and t_1/2_ (10.11 h) implied that Simvastatin release from this drug delivery system successfully bypassed first pass effect and provided higher bioavailability. Manufacturing of dMNPs is a challenging task and time-consuming process that can be optimized by tuning process variables. For future prospects, we would suggest carrying out efficacy and penetration studies of this dMNP onto human skin. Moreover, these drug delivery systems can be employed in disease-induced animal models. 

## Figures and Tables

**Table 1:** Composition of dissolvable microneedle patches (F1–F26).

Polymers (%)	F1	F2	F3	F4	F5	F6	F7	F8	F9	F10	F11	F12	F13	F14	F15	F16	F17	F18	F19	F20	F21	F22	F23	F24	F25	F26
TC	0.5	1	1.5	2	-	-	-	-	0.5	0.5	1	1	1	1	2	2	2	2	1	2	1	2	1	1	2	2
PVP	-	-			5	-	10	-	10	-	5	-	10	-	5	-	10	-	5	5	10	10	5	10	10	5
PVA	-	-	-	-	-	5	-	10	-	10	-	5	-	10	-	5	-	10	5	5	10	10	10	5	5	10

**Table 2:** Thickness results of Parafilm M layers.

Sr. No.	Description	Thickness (mm)	Thickness (mm)	Thickness (mm)	Average Thickness (mm)
1	Thickness of two glass slides	2.20	2.21	2.19	2.20
2	Thickness of slides with 9 layers of Paraffin M films	3.48	3.47	3.46	3.47
3	Thickness of one Paraffin film M layer	0.142	0.140	0.141	0.141

**Table 3:** FTIR peaks and corresponding functional groups.

Polymer	Functional Groups	Peaks
PVP	C=O	1642.13 cm^−1^
	CH_2_	2981.67 cm^−1^
	CH	1290.14 cm^−1^
	CH	1425.13 cm^−1^
	C-N	1313.11 cm^−1^
PVA	OH	3296 cm^−1^
	CH	2921 cm^−1^
	OH	1411 cm^−1^
	C-O-	1090 cm^−1^
Thiolated Chitosan	OH	3352.5 cm^−1^
	NH	3201 cm^−1^
	Acylamino	1631 cm^−1^
	Amino group with thioglycolic acid	1605 cm^−1^
Simvastatin	–OH	3550 cm^−1^
	–CH	3747 cm^−1^
	–C-O-	2950 cm^−1^
	–C=O	1740 cm^−1^
Simvastatin loaded dMNP (F26)	–C-O-	1644.35 cm^−1^
	–CH	2950 cm^−1^

**Table 4:** Release results of reported dMNPs.

Sr. No.	Drug Release Behaviour	Reference
1	In vitro drug release was found to be 65% in 48 h.	Habib R. et al. (2022) [37]
2	dMNPs made of PVP matrix resulted in drug release of 80%.	Mao J. et al. (2020) [51]
3	Drug release reported in this study is 82.5%.	Ahmad Z. et al. (2020) [35]
4	Drug release was found to be 82.7%.	Yavuz B. et al. (2020) [52]
5	Bovine serum albumin release was up to 95% from MNPs.	Chen M-C. et al. (2012) [53]

**Table 5:** Kinetic modelling of release data.

Models	Parameters	F26	Simvastatin Solution
Zero Order	R^2^	0.989	0.91
	T_25_	14.842	24.728
	T_50_	29.683	49.457
	T_75_	44.525	74.185
First Order	R^2^	0.8916	0.8732
	T_25_	11.171	22.500
	T_50_	26.915	41.213
	T_75_	53.830	62.425
Higuchi Model	R^2^	0.89	0.93
	T_25_	6.041	22.500
	T_50_	24.163	54.213
	T_75_	54.367	72.425
Korsemeyer Peppas	R^2^	0.977	0.93
	N	1.436	0.987

**Table 6:** Pharmacokinetic parameters of Simvastatin after administration of dMNP and oral solution.

Parameters	F26 (Mean ± S.D)	Table Simva Solution (Mean ± S.D.)
C_max_ (µg/mL)	1.97 ± 0.002	2.55 ± 0.089
t_max_ (h)	9 ± 0.453	3 ± 0.786
t_1/2_ (h)	10.11 ± 0.342	2.31 ± 1.23
AUC 0-t (µg/mL·h)	46.243 ± 0.123	14.20 ± 0.871
AUMC _0-∞_ (µg/mL·h^2^)	48.941 ± 0.223	15.19 ± 0.342
MRT (h)	19.91 ± 0.334	5.91 ± 0.233
Clearance (mg)/(μg/mL)/h	0.408 ± 0.033	1.314 ± 0.223

## Data Availability

The data presented in this study are available on request from the corresponding author.
